# Assessment of Weight Management Practices among Adults in the United Arab Emirates

**DOI:** 10.1155/2017/1050749

**Published:** 2017-09-24

**Authors:** Amita Attlee, Nour Atmani, Viktor Stromtsov, Fatima Ali, Rim Tikarly, Sarah Ryad, Ghada Salah, Hayder Hasan, Reyad Obaid

**Affiliations:** ^1^Department of Clinical Nutrition and Dietetics, College of Health Sciences, University of Sharjah, Sharjah, UAE; ^2^Nutrition and Health Department, College of Food and Agriculture, United Arab Emirates University, P.O. Box 15551, Al Ain, UAE; ^3^Research Institute for Medical and Health Sciences, University of Sharjah, Sharjah, UAE

## Abstract

With a rise in global incidence of overweight and obesity, the number of patients seeking weight management (WM) advice is likely to increase. Our aim was to explore the prevalence of WM practices and investigate association of WM goals with sociodemographic variables and practices among United Arab Emirates (UAE) adults. An exploratory, cross-sectional research was conducted on 1275 adult males and females, residing in UAE. A structured questionnaire was administered. WM goals to lose/maintain/gain weight were reported in 88.3% participants. WM goals were significantly associated with age, sex, marital status, education, current body weight perception, and medical condition. Out of 21 selected WM practices, popular strategies included increasing physical activity (52.9%), eating less fat (51.1%), consuming fewer calories (43.3%), joining gym (27.5%), skipping meals (26.1%), and consuming natural herbs and teas (20.7%). Visiting dietitian (12.3%) ranked ninth in the order of preference. Males focused on physical activity, gyms, and wellness centers and females on calories counting, dietitian visits, meals replacement, skipping meals, and natural herbs/teas. Married adults reported eating less fat (54.3% versus 47.3%, *p* = 0.020); singles opted calories counting, gyms, and meals replacement. Frequent referral sources were friends (37.8%) and Internet (32.1%). Most UAE adults had WM goals that were associated with sociodemographic variables and WM practices. Awareness about the ill-effects of unhealthy WM practices and importance of dietitian's consultation are imperative.

## 1. Introduction

American Medical Association (2013) resolved that obesity should be considered as a chronic medical disease state [1]. With the global incidence of overweight and obesity among adults continuing to rise, the number of patients seeking advice for weight management (WM) is likely to increase. Recent estimates on the prevalence of overweight and obesity are astounding in Arabian Gulf countries particularly in Kuwait, Qatar, Saudi Arabia, and Bahrain where between two-thirds to three-quarters of adults and adolescents are overweight or obese [2]. Approximately 75% of people in the UAE are obese or overweight [3]. On the other hand, poor nutrition results in undernutrition that is evident as the state of underweight. Nearly 3.9% of Emirati women were found to be underweight according to the National Nutrition Survey in the UAE [4]. Significant weight loss in people tempts them to try varied strategies of weight gain. Attempts to lose, maintain, or gain weight are common practices among individuals. However, the strategies to achieve these goals vary. Some prefer diets prescribed by dietitians, while others follow “popular” or “fad” diets encouraging irrational and, sometimes, unsafe practices [5]. A plethora of WM services are available in the UAE ranging from weight-loss/fitness centers to bariatric surgeries; however, statistics on their usage and client registries are not yet established.

Dietitians take the lead in the promotion of public health nutrition through the blend of scientific knowledge and understanding of social and cultural factors that influence what people eat [6]. However, individuals typically select WM practices that they feel most comfortable trying, fit into their budget, and are reasonably likely to be successful. The variety of practices adopted for WM ranges from commercial to medical and surgical approaches [7].

Reduction in total caloric intake, skipping meals, fasting ≥24 hours, taking diet pills or diuretics, and joining weight-loss programs [8]; increasing physical activity, eating diet foods or products, drinking a lot of water, and following a special diet [9]; or using meal replacements are common strategies utilized to achieve the desired WM goals. Moreover, bariatric surgery has been demonstrated to be the most effective and long term treatment for individuals with severe obesity or moderate obesity complicated by comorbid conditions that is not responsive by nonsurgical approaches [7]. Making diet and lifestyle changes can be difficult, turning many people to dietary supplements for weight loss in the hope that these products will help them more easily achieve their weight-loss goals. These supplements encompass a wide variety of products and come in a variety of forms, including capsules, tablets, liquids, powders, and bars [10]. Using pharmacological agents as adjuvant therapy after lifestyle intervention to maintain the weight loss achieved is popular for weight loss and prevention of regain [11]. For underweight individuals, appetite stimulants and commercial drugs are accessible for weight gain and optimum body shape [12].

Potentially harmful weight control practices were reported in females including excessive exercise, starvations, purging, laxatives, slimming tablets, and smoking for weight control, while males used excessive exercise, starvation, and smoking to lose or maintain weight [13].

Different WM practices adopted by individuals are associated with several factors such as age and gender [8, 14, 15], education level [16], marital status [17], socioeconomic status [18], peer pressure, and health issues. Women's but not men's marital roles appear to influence their perceived and desired weight, suggesting that weight management interventions should be sensitive to both marital status and gender differences [17]. Further, high income individuals were more likely to recognize being overweight and were more likely to attempt weight control [18]. However, unhealthy WM strategies may lead to stunted growth, nutrient deficiencies, infections, clinically significant eating disorders, increased risks of osteoporosis, and anemia [19]. There is a lack of evidence on WM practices and their determinants among adults residing in the UAE.

The aim of this study was to explore the prevalence of WM practices and investigate the association of WM goals with sociodemographic variables and practices among adult residents in the UAE.

## 2. Material and Methods

An exploratory study with a cross-sectional research design was conducted. Sample size was calculated based on the estimate of 70% prevalence of overweight and obesity in UAE adult population [3] with 90% confidence. A total of 1275 males and females aged 18 years or above residing in the seven Emirates of the UAE (Abu Dhabi, Dubai, Sharjah, Ajman, Fujrairah, Ras Al Khaimah, and Umm Al Quwain) participated in the study. These participants were selected from a free living population in UAE through convenience sampling method from different malls, hyper- and supermarkets, institutions, and neighborhood. The research team members approached the prospective participants and briefed them on the purpose of study and if they expressed interest, they were requested to sit in a calm area with the researcher for further formalities and participation. A structured questionnaire was adapted [16] and modified for clarity and cultural applicability after being pilot tested on 12 adults who were not included in the study sample. It consisted of sections to collect information on sociodemographic profiles, body weight perception and medical conditions, WM goals and practices, sources of referrals for adoption of WM practices, and duration and outcomes of adopted WM practices. Twenty-one common WM practices were included that could cover the broad spectrum WM goals of losing, maintaining, or gaining body weight. Pregnant women were excluded from participation in this study. The protocol was approved by the research committee, Clinical Nutrition and Dietetics Department, College of Health Sciences, University of Sharjah (number 4/11/2013). Informed written consent was taken before administering the questionnaire and participant anonymity was maintained.

Data analysis was carried out using the Statistical Package for the Social Sciences Software (SPSS version 17.0, IBM, USA). Descriptive statistics of frequencies and percentages were used to determine the prevalence of WM among adults in the UAE. Chi-square was used to assess the association of independent variables such as age, sex, nationality, marital status, income, and WM practices and the dependent variable, WM goals. Age cut-off at 45 years was considered since the trend of overweight and obesity changes around 45 years of age in Gulf Cooperation Council countries [20, 21]. The data was tested for significance at *p* < 0.05.

## 3. Results

At the time of survey period, most of the participants reported having a WM goal. Out of 1275 participants, 1126 (88.3%) participants had a WM goal and 149 (11.7%) participants neither were concerned about their body weights nor reported any WM goal at the time of survey. Out of 1126 (those who had a WM goal), 624 (55.4%) of them were trying to lose weight and 408 (36.2%) reported having a WM goal to maintain weight, while 94 (8.4%) participants were attempting to gain weight. Results below are presented for those 1126 participants who had a WM goal.

Sociodemographic characteristics (Table 1) showed that the majority of participants (83.1%) were below the age of 45 years; 58.4% were females and 41.6% males. More than half (53.1%) of the participants were married and the rest (46.9%) were singles. Emirati nationals constituted 15.9% of the 1126 participants, while remaining 84.1% were expatriates from other nationalities. Out of 1126 participants, 897 (79.7%) had completed higher education; almost two-thirds (736/1126) of the participants reported monthly income of less than AED 20,000 (equivalent to USD 5,450). All sociodemographic variables, except nationality and monthly income, were found to be significantly associated with the WM goals. Comparatively, a significant higher proportion of younger adult population (*p* = 0.001), females (*p* < 0.001), and married (*p* < 0.001) adults reported having WM goals. Higher education status was also found to be significantly associated with the WM goals (*p* = 0.014).

It was interesting to note that the WM goals of the participants were significantly associated with their perception of current body weight (*p* < 0.001). Overall, 595 (52%) participants perceived themselves with “normal weight” followed by 417 (37%) as “overweight” and 66 (5.9%) as “underweight” and 58 (5.1%) perceived their current body weight as “obese.” Comparatively, majority of the participants with the goal to lose weight perceived their body weight as “overweight” (382/624; 61.2%); majority of those who aimed to maintain weight perceived themselves as “normal” weight (359/408; 88%) and most of those who had the goal to gain weight perceived that they were “underweight” (46/94; 48.9%).

There was a significant association in the WM goal and medical condition of the participants. High cholesterol, hypertension, and diabetes mellitus were self-reported in 12.5% (*n* = 141; *p* < 0.001), 11.5% (*n* = 129; *p* < 0.001), and 10.2% (*n* = 115; *p* = 0.04) participants, respectively. Almost three-fourths (68.7%–76.7%) of the participants with positive self-reporting of any of these medical conditions had a WM goal of losing weight; 20.2%–25.2% had a goal of weight maintenance; and 4.3%–7.4% participants aimed towards gaining weight.

Table 2 shows the distribution of 1126 participants according to the selected 21 WM practices adopted by them during the study period. Six popular strategies emerged including increasing physical activity by enhancing movements of skeletal muscles, not necessarily following an organized regime (52.9%), eating less fat (51.1%), consuming fewer calories (43.3%), joining gym to increase physical activity through exercises in a controlled environment (27.5%), skipping meals (26.1%), and consuming natural herbs and teas (20.7%). Visiting a dietitian was not reported as a common WM practice with 12.3% (138/1126) participants stating that they visited a dietitian, ranking ninth in the order of preference. Fourteen out of 21 selected WM practices were found to have a significant association with the WM goals of the participants. On the other hand, practices such as joining gyms and wellness centers, supplements use and pharmacotherapy, and bariatric surgery as well as subscribing special diets were independent of the WM goal.

Further, significant associations between sociodemographic variables (sex, marital status, and nationality) and WM practices among UAE adults are presented in Table 3. As evident, a significantly higher proportion of males than females reported to have increased physical activity (57.5% versus 49.7%, *p* = 0.011) and joined gyms (34.8% versus 22.3%, *p* < 0.001) and wellness centers (11.8 versus 7.6%, *p* = 0.022) for management of their weights. In contrast, more females than males either consumed fewer calories (47.0% versus 38.2%, *p* = 0.004), skipped meals (28.6% versus 22.6%, *p* = 0.028), consumed natural herbs and teas (23.3% versus 17.1%, *p* = 0.014), visited a dietitian (14.1% versus 9.6%, *p* = 0.027), or counted calories (8.2% versus 3.4%, *p* = 0.001).

Marital status was also found to be significantly associated with certain WM practices (Table 3). While married participants reported eating less fat (54.3% versus 47.3%, *p* = 0.020) for managing their weights, significantly higher proportions of participants with single marital status reported either focusing on their calories consumption, joining gyms, or meal replacement compared to their married counterparts.

Besides, Table 3 highlights that a significantly higher proportion of UAE nationals (17.3%) reported visiting the dietitian for managing weights in contrast to 11.3% expatriate participants (*p* = 0.024). On the other hand, 7.2% expatriates in comparison with 1.1% nationals reported counting calories to manage their body weights (*p* = 0.001).

Participants reported different sources of referrals for the adoption of their WM practices: friends (37.8%), Internet (32.1%), dietitians (23.5%), television (23.1%), gym instructors (23.0%), and doctors/physicians (21.0%). Lesser referred sources included relatives (18.7%), magazines (18.4%), newspapers (12.9%), radio (9.5%), and neighbors (6.1%).

Duration of adoption of current WM practices varied in the 1126 participants: while 36.8% (*n* = 414) reported that they adopted the current strategies since less than last 3 months, 27.8% (*n* = 313) were practicing the reported WM strategies for more than 12 months, followed by 22.4% (*n* = 252) for the last 3–6 months. Only 11.5% (*n* = 130) participants reported that their current WM practices were adopted for the last 6–12 months from the survey period. However, the rest (*n* = 17) did not respond to the duration of adoption of WM practices.

The outcome of adopted WM practices was reported in terms of the change in body weight during the period by 1095 out of 1126 participants. Out of those who responded, 63.6% (696/1095) reported less than 5 kg change in weight, 28.9% (317/1095) between 5 and 10 kg, 5.3% (58/1095) more than 10–15 kg, and 2.2% (24/1095) reported a change of more than 15 kg in body weight.

## 4. Discussion

Our results emphasized that the practices were associated with the WM goals in adults. Most common WM practices found in the present study included increasing physical activity, eating less fat, and consuming fewer calories. Lin et al. (2013) reported exercise to be a more common method than switching to foods with lower calories for losing weight [9]. Further, Kruger et al. (2004) found that only one-third of all those people who were trying to lose weight reported eating less calories and exercising more as the strategies [8]. Visiting dietitian was not a common WM practice and ranked ninth in the order of participants' preferences in our study. Spikmans et al. (2003) reported that one in three patients with diabetes attending the out-patient clinics skipped one or more visits to their dietitian [22]. Primary care physicians continue to believe that providing nutrition counseling is within their realm of responsibility; patients seemed to expect dietary counseling from their physician rather than from dietitians [23], and most patients reported apprehension and doubt towards the usefulness of dietary advice from dietitians [22].

The present study highlighted that a significantly higher percentage of females (58.4%) than males (41.6%) had a WM goal. Similar trend was reported by Kruger et al. (2004), wherein 24% of men and 38% of women were trying to lose weight [8]. Sex-wise analysis of our study showed that more females than males counted or reduced calories, visited dietitian, and consumed natural herbs and teas. Skipping meals was also reported more in women than men. Serdula et al. (1994) revealed that the women were more likely than men to report counting calories, belonging to an organized weight-loss program, taking special supplements, or taking diet pills. The use of these specific weight control practices was lower for those who were trying to maintain weight [16]. Kruger et al. (2004) also supported that more women than men adopted eating fewer calories, eating less fat, eating food supplements, joining a weight-loss program, taking diet pills and diuretics, or fasting as the strategies to lose weight [8].

Our study revealed that the WM goal was independent of income. However, income was a significant predictor of whether or not overweight or obese adults receive weight-loss advice [24]. Though the UAE nationals constitute about 11% of the country's population [25], it was evident that a higher proportion of UAE nationals visited a dietitian than the UAE nonnationals. This might be attributed to the fact that UAE nationals have access to free health care and would visit the dietitian to get appropriate WM strategies. Refusal of insurance companies to reimburse dietetic counseling continues to be a barrier for the utilization of these services [26]. Evidence from United States clearly states that the utilization of dietetic services has increased ever since the policy of reimbursement for dietary counseling by registered dietitian under multidisciplinary approach as a medical benefit was introduced [27].

Overall, more married adults reported having WM goals of either losing weight or maintaining weight. In contrast, significantly higher number of participants with single marital status had the WM goal to gain weight. Further, more participants with single marital status practiced counting calories, joining gym, and consuming replacement meals than those married. Earlier, Klos and Sobal (2013) analyzed that marital status was unrelated to WM approach, except that divorced or separated women were more likely to have intentionally lost weight within the past year as compared to never married women. Additionally, never married men were more likely to be attempting to prevent weight gain than married or cohabiting men [17].

Most referred source of information for a WM approach in the present study was “friends” and “Internet.” This might have been one of the reasons for the participants to have followed unhealthy WM practices, as information might be distorted and incorrect. A study from the Gulf involving Emirati and Omani adults with diabetes revealed that one-fourth of the patients relied on informal networks such as friends and family for nutrition information [28].

The present study had some inherent limitations due to logistic reasons. The sampling was based on convenience rather than randomization. However, it was ensured that the participants were recruited from all the seven Emirates in proportion aligning the national population distribution. Further, information was gathered on perceived and not actual body weights. Yet, interesting findings emerged indicating that majority of the participants perceived themselves as having “normal” body weights. This may be one of the barriers for adults in losing weight in an otherwise “overweight/obese” country. Considering an exploratory nature of this study, it highlighted common WM practices and important sociodemographic determinants associated with WM goals among adults residing in the UAE.

## 5. Conclusion

Adults residing in the UAE generally have a WM goal and they adopt a variety of WM practices to achieve their goals. Unhealthy practices for WM such as skipping meals, following a popular diet, and pharmacotherapy are witnessed among the UAE adult population while visiting the dietitian is not a common practice. This poses important concerns on the nutrition knowledge and motivation of the individuals and competence of the dietitians as well as the cost and reimbursement policy of the dietitian's consultation by health insurance.

Awareness is required in the UAE about the ill-effects of unhealthy WM practices and importance of dietitian's consultation to achieve the desired WM goals in a safe and healthy way. As evident in other countries, a health insurance policy to cover dietitians' visits within a multidisciplinary health care team might be a prospective approach to scale up evidence based WM practices among adult population in the UAE.

## Figures and Tables

**Table 1:** Associations between sociodemographic characteristics and weight management goals among UAE adults (Chi-square test).

Sociodemographic variable		Weight management goal			*p* value
	Total *N* = 1126	Lose weight *N* = 624	Maintain weight *N* = 408	Gain weight *N* = 94	
	*N* (%)	*N* (%)	*N* (%)	*N* (%)	
Age group (years)					
<45	936 (83.1)	499 (80)	348 (85.3)	89 (94.7)	0.001
≥45	190 (16.9)	125 (20)	60 (14.7)	5 (5.3)	
Sex					
Male	468 (41.6)	219 (35.1)	197 (48.3)	52 (55.3)	<0.001
Female	658 (58.4)	405 (64.9)	211 (51.7)	42 (44.7)	
Marital status					
Single	528 (46.9)	277 (44.4)	182 (44.6)	69 (73.4)	<0.001
Married	598 (53.1)	347 (55.6)	226 (55.4)	25 (26.6)	
Nationality					
UAE national	179 (15.9)	102 (16.3)	61 (15.0)	16 (17.0)	0.792
UAE nonnational	947 (84.1)	522 (83.7)	347 (85.0)	78 (83.0)	
Education					
Up to high school	229 (20.3)	146 (23.4)	65 (15.9)	18 (19.1)	0.014
Graduate or above	897 (79.7)	478 (76.6)	343 (84.1)	76 (80.9)	
Income (AED)					
<20,000	736 (65.4)	397 (63.6)	273 (66.9)	66 (70.2)	0.321
>20,000	390 (34.6)	227 (36.4)	135 (33.1)	28 (29.8)	

**Table 2:** Associations between weight management practices and weight management goals among UAE adults (Chi-square test).

Weight management practice		Weight management goal			*p* value
	Total	Lose weight	Maintain weight	Gain weight	
	*N* (%)	*N* (%)	*N* (%)	*N* (%)	
Increasing physical activity					
Yes	596 (52.9)	344 (55.1)	214 (52.5)	38 (40.4)	0.028
No	530 (47.1)	280 (44.9)	194 (47.5)	56 (59.6)	
Eating less fat					
Yes	575 (51.1)	363 (58.2)	194 (47.5)	18 (19.1)	<0.001
No	551 (48.9)	261 (41.8)	214 (52.5)	76 (80.9)	
Consuming fewer calories					
Yes	488 (43.3)	335 (53.7)	142 (34.8)	11 (11.7)	<0.001
No	638 (56.7)	289 (46.3)	266 (65.2)	83 (88.3)	
Joining gym					
Yes	310 (27.5)	165 (26.4)	113 (27.7)	32 (34.0)	0.305
No	816 (72.5)	459 (73.6)	295 (72.3)	62 (66.0)	
Skipping meals					
Yes	294 (26.1)	202 (32.4)	90 (22.1)	2 (2.1)	<0.001
No	832 (73.9)	422 (67.6)	318 (77.9)	92 (97.9)	
Taking natural herbs and teas					
Yes	233 (20.7)	141 (22.6)	87 (21.3)	5 (5.3)	0.001
No	893 (79.3)	483 (77.4)	321 (78.7)	89 (94.7)	
Taking special supplements for weight management					
Yes	193 (17.1)	110 (17.6)	69 (16.9)	14 (14.9)	0.797
No	933 (82.9)	514 (82.4)	339 (83.1)	80 (85.1)	
Following a popular (fad) diet					
Yes	149 (13.2)	100 (16.0)	45 (11.0)	4 (4.3)	0.002
No	977 (86.8)	524 (84.0)	363 (89.0)	90 (95.7)	
Visiting a dietitian					
Yes	138 (12.3)	96 (15.4)	31 (7.6)	11 (11.7)	0.001
No	988 (87.7)	528 (84.6)	377 (92.4)	83 (88.3)	
Fasting					
Yes	118 (10.5)	81 (13.0)	37 (9.1)	0 (0.00)	<0.001
No	1008 (89.5)	543 (87.0)	371 (90.9)	94 (100.0)	

Joining wellness centre					
Yes	105 (9.3)	62 (9.9)	31 (7.6)	12 (12.8)	0.220
No	1021 (90.7)	562 (90.1)	1021 (90.7)	82 (87.2)	
Replacement foods					
Yes	76 (6.7)	32 (5.1)	31 (7.6)	13 (13.8)	0.005
No	1050 (93.3)	592 (94.9)	377 (92.4)	81 (86.2)	
Counting calories					
Yes	70 (6.2)	50 (8.0)	17 (4.2)	3 (3.2)	0.020
No	1056 (93.8)	574 (92.0)	391 (95.8)	91 (96.8)	
Using pharmacotherapy					
Yes	56 (5.0)	32 (5.1)	21 (5.1)	3 (3.2)	0.708
No	1070 (95.0)	592 (94.9)	387 (94.9)	91 (96.8)	
Consuming more calories					
Yes	53 (4.7)	7 (1.1)	12 (2.9)	34 (36.2)	<0.001
No	1073 (95.3)	617 (98.9)	396 (97.1)	60 (63.8)	
Taking appetite stimulants					
Yes	39 (3.5)	6 (1.0)	15 (3.7)	18 (19.1)	<0.001
No	1087 (96.5)	618 (99.0)	393 (96.3)	76 (80.9)	
Purging (vomiting)					
Yes	37 (3.3)	30 (4.8)	6 (1.5)	1 (1.1)	0.006
No	1089 (96.7)	594 (95.2)	402 (98.5)	93 (98.9)	
Eating more fat					
Yes	37 (3.3)	5 (0.8)	6 (1.5)	26 (27.7)	<0.001
No	1089 (96.7)	619 (99.2)	402 (98.5)	68 (72.3)	
Subscribing a special diet					
Yes	34 (3.0)	20 (3.2)	10 (2.5)	4 (4.3)	0.602
No	1092 (97.0)	604 (96.8)	398 (97.5)	90 (95.7)	
Others					
Yes	11 (1.0)	5 (0.8)	5 (1.2)	1 (1.1)	0.792
No	1115 (99.0)	619 (99.2)	403 (98.8)	93 (98.9)	
Bariatric surgery					
Yes	6 (0.5)	4 (0.6)	2 (0.5)	0 (0.00)	0.721
No	1120 (99.5)	620 (99.4)	406 (99.5)	94 (100.0)	

**Table 3:** Significant associations between sociodemographic variables (sex, marital status, and nationality) and weight management practices among UAE adults (Chi-square test).

Weight management practice	Total *N* = 1126	Males *N* = 468	Females *N* = 658	*p* value
	*N* (%)	*N* (%)	*N* (%)	

Increasing physical activity				
Yes	596 (52.9)	269 (57.5)	327 (49.7)	0.011
No	530 (47.1)	199 (42.5)	331 (50.3)	
Consuming fewer calories				
Yes	488 (43.3)	179 (38.2)	309 (47.0)	0.004
No	638 (56.7)	289 (61.8)	349 (53.0)	
Joining gym				
Yes	310 (27.5)	163 (34.8)	147 (22.3)	<0.001
No	816 (72.5)	305 (65.2)	511 (77.7)	
Skipping meals				
Yes	294 (26.1)	106 (22.6)	188 (28.6)	0.028
No	832 (73.9)	362 (77.4)	470 (71.4)	
Taking natural herbs and teas				
Yes	233 (20.7)	80 (17.1)	153 (23.3)	0.014
No	893 (79.3)	388 (82.9)	505 (76.7)	
Visiting a dietitian				
Yes	138 (12.3)	45 (9.6)	93 (14.1)	0.027
No	988 (87.7)	423 (90.4)	565 (85.9)	
Joining wellness centre				
Yes	105 (9.3)	55 (11.8)	50 (7.6)	0.022
No	1021 (90.7)	413 (88.2)	608 (92.4)	
Counting calories				
Yes	70 (6.2)	16 (3.4)	54 (8.2)	0.001
No	1056 (93.8)	452 (96.6)	604 (91.8)	

Weight management practice	Total *N* = 1126	Single *N* = 528	Married *N* = 598	*p* value
	*N* (%)	*N* (%)	*N* (%)	

Eating less fat				
Yes	575 (51.1)	250 (47.3)	325 (54.3)	0.020
No	551 (48.9)	278 (52.7)	273 (45.7)	
Consuming more calories				
Yes	53 (4.7)	32 (6.1)	21 (3.5)	0.049
No	1073 (95.3)	496 (93.9)	577 (96.5)	
Joining gym				
Yes	310 (27.5)	176 (33.3)	134 (22.4)	<0.001
No	816 (72.5)	352 (66.7)	464 (77.6)	
Counting calories				
Yes	70 (6.2)	54 (10.2)	16 (2.7)	<0.001
No	1056 (93.8)	474 (89.8)	582 (97.3)	
Replacement meals				
Yes	76 (6.7)	47 (8.9)	29 (4.8)	0.009
No	1050 (93.3)	481 (91.1)	569 (95.2)	

Weight management practice	Total *N* = 1126	UAE National *N* = 179	Non-UAE National *N* = −947	*p* value
	*N* (%)	*N* (%)	*N* (%)	

Visiting a dietitian				
Yes	138 (12.3)	31 (17.3)	107 (11.3)	0.034
No	988 (87.7)	148 (82.7)	840 (88.7)	
Counting calories				
Yes	70 (6.2)	2 (1.1)	68 (7.2)	0.001
No	1056 (93.8)	177 (98.9)	879 (92.8)	
